# The oldest species of *Peltoperleidus* (Louwoichthyiformes, Neopterygii) from the Middle Triassic (Anisian) of China, with phylogenetic and biogeographic implications

**DOI:** 10.7717/peerj.12225

**Published:** 2021-09-29

**Authors:** Guang-Hui Xu

**Affiliations:** 1 Key Laboratory of Vertebrate Evolution and Human Origins of Chinese Academy of Sciences, Institute of Vertebrate Paleontology and Paleoanthropology, Chinese Academy of Sciences, Beijing, China; 2 CAS Center for Excellence in Life and Paleoenvironment, Beijing, China

**Keywords:** Osteology, Phylogeny, Louwoichthyiformes, Neopterygii, Triassic

## Abstract

The previously alleged ‘perleidid’ genus *Peltoperleidus* is a stem-neopterygian fish taxon with two or three horizontal rows of notably deepened flank scales. Until recently, members of this genus were known only from the Ladinian (late Middle Triassic) or near the Anisian/Ladinian boundary (~242 Ma) in southern Switzerland and northern Italy. Here, I report the discovery of a new species of the genus, *Peltoperleidus asiaticus* sp. nov., based on three well-preserved specimens from the Anisian (early Middle Triassic, ~244 Ma) of Luoping, eastern Yunnan, China. The discovery extends the geological range of *Peltoperleidus* by approximately two million years and documents the first record of the genus in Asia. Similar to its relatives (represented by *P. macrodontus*) from Europe, *P. asiaticus* sp. nov. is likely a small-sized durophagous predator with dentition combining grasping and crushing morphologies. Results of a cladistic analysis unite four species of *Peltoperleidus* as a monophyletic group within the Louwoichthyiformes, and suggest that the presence of two horizontal rows of notably deepened scales was independently evolved in *Peltoperleidus* and another stem-neopterygian taxon *Altisolepis*. *P. asiaticus* sp. nov. is nested at the base of *Peltoperleidus*, and a new family Peltoperleididae is proposed for the genus, contrasting the previous placement of *Peltoperleidus* in the poorly defined, paraphyletic ‘Perleididae’. Comparative studies of the basal peltoperleidid from China with its younger relatives from Europe provide new insights into the evolutionary origin and paleogeographic distribution of this clade.

## Introduction

Neopterygii (Teleostei, Holostei and closely related fossil taxa) is the largest group of ray-finned fishes today, exhibiting high morphological and taxonomic diversity (*e.g*., Gardiner, Maisey & Littlewood, 1996; Grande & Bemis, 1998; Cavin, 2010; Grande, 2010; Sallan, 2014; Friedman, 2015; Arratia, 2015; Nelson, Grande & Wilson, 2016; López-Arbarello & Sferco, 2018; Xu, 2021). This group underwent a rapid radiation in the aftermath of the end-Permian mass extinction (*e.g*., Benton et al., 2013; Romano et al., 2016; Clarke & Friedman, 2018). Fossil records of stem-neopterygian taxa are particularly rich from the Triassic deposits in almost all continents except Antarctica, and they have long attracted the attention of palaeoichthyologists interested in the early diversification of this clade (*e.g*., Brough, 1931, 1939; Stensiö, 1932; Wade, 1935; Lehman, 1952; Schaeffer, 1956; Gardiner, 1960; Patterson, 1973; Hutchinson, 1973a, b; Griffith, 1977; Bürgin et al., 1991; Tintori & Sassi, 1992; Lombardo, 2001; Bürgin & Herzog, 2002; Mutter, 2004, 2005; Lombardo & Brambillasca, 2005; López-Arbarello & Zavattieri, 2008; Sun et al., 2009, 2012; Geng et al., 2012; Xu, Gao & Coates, 2015; Xu, Zhao & Shen, 2015, Xu, Ma & Zhao, 2018; Cartanyà et al., 2015, 2019; Xu & Ma, 2016; Marramà et al., 2017; Wen et al., 2019; Xu, 2020a, 2020b; Ma, Xu & Geng, 2021).

The extinct genus *Peltoperleidus* is a stem-neopterygian taxon from the Ladinian (late Middle Triassic) or near the Anisian/Ladinian boundary in southern Switzerland and northern Italy (Bürgin et al., 1991; Herzog, 2001; Mutter & Herzog, 2004). This genus has two or three horizontal rows of notably deepened flank scales, showing an intermediate condition between perleidids and peltopleurids (from that its name was derived). The type species, *P. ducanensis*, was named by Bürgin et al. (1991) on the basis of three specimens from the Ladinian of Canton Grisons, eastern Switzerland. Bürgin (1992) and Herzog (2001) referred five additional species to this genus, but Mutter & Herzog (2004) considered three of them (*P. obristi*, *P. macrodontus* and *P. triseries*) as valid, and moved the remaining two into another stem-neopterygian genus *Altisolepis*. Both *Peltoperleidus* and *Altisolepis* were originally placed in the Perleididae (Bürgin, 1992; Herzog, 2001; Mutter & Herzog, 2004) and *Altisolepis* was later referred to the Peltopleuridae (Sun et al., 2015). However, these taxonomic hypotheses are not based on phylogenetic analyses.

**Figure 1:**
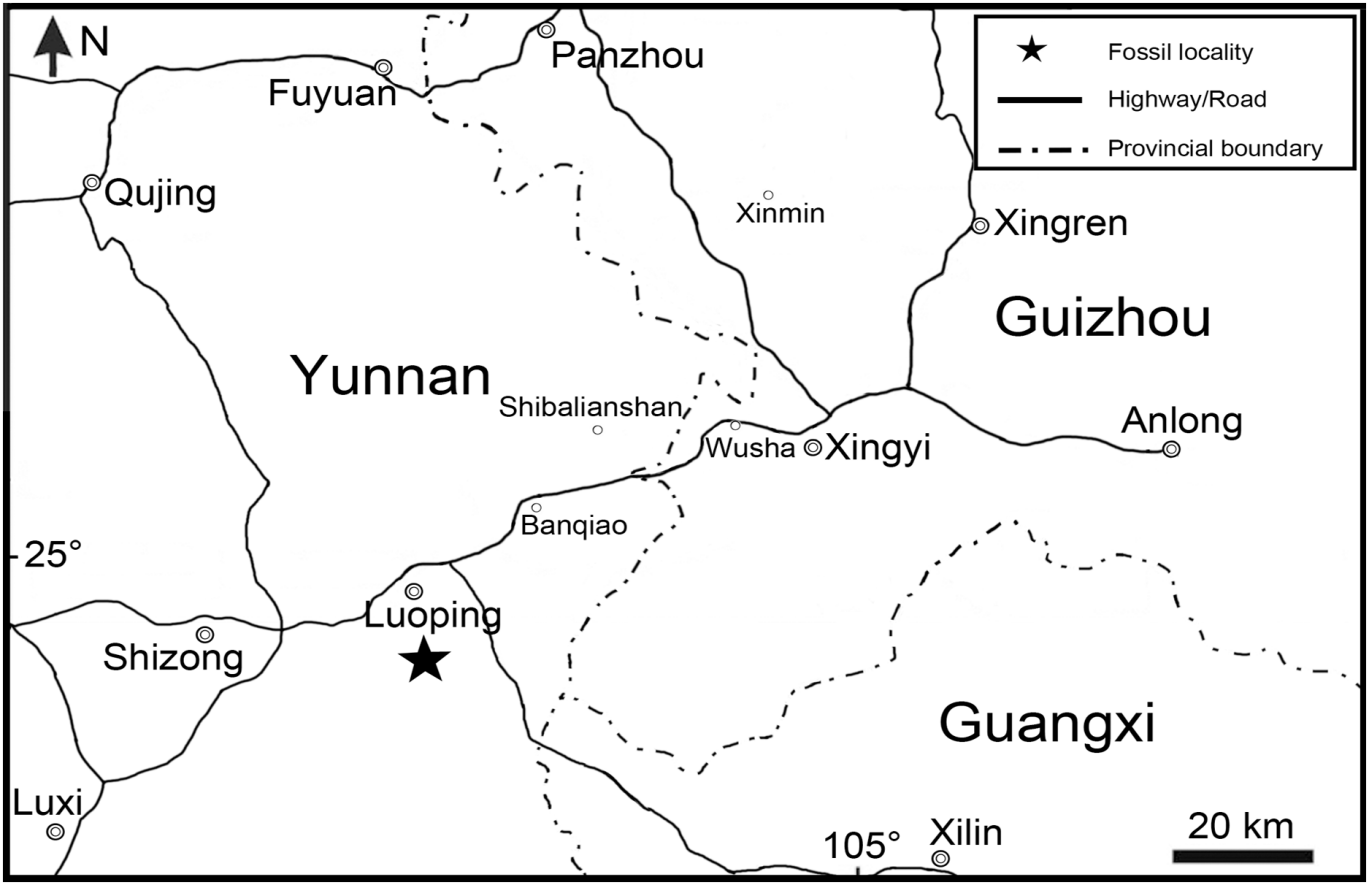
Fossil locality. Map showing the fossil locality of *Peltoperleidus asiaticus* sp. nov.

Here, I report the discovery of a new species of *Peltoperleidus* based on three well-preserved specimens from the Second (Upper) Member of the Guanling Formation exposed in Luoping, eastern Yunnan, China (Fig. 1). The discovery documents the first record of *Peltoperleidus* outside Europe, significantly adding our knowledges on the paleogeographic distribution of the genus. In addition, the superb preservation of the skeletons of the new species provides us more comprehensive information on the anatomical diversity of the genus. The aim of this paper is to describe the morphology of the new species of *Peltoperleidus* and to discuss the affinities of *Peltoperleidus* and *Altisolepis* with other early neopterygian clades using cladistic approaches.

Along with the new species of *Peltoperleidus*, other taxa from the same fossiliferous level in Luoping localities include plants, invertebrates, marine reptiles, and other groups of bony fishes; the whole fossil assemblage has been referred to the Luoping Biota (*e.g*., Zhang et al., 2009; Sun et al., 2009, 2015; Tintori et al., 2010; López-Arbarello et al., 2011; Wu et al., 2011; Lin et al., 2011; Lombardo et al., 2011; Xu & Wu, 2012; Geng et al., 2012; Wen et al., 2019; Feldmann et al., 2012; Huang et al., 2013; Benton et al., 2013; Xu, Zhao & Coates, 2014, Xu, Ma & Zhao, 2018; Xu & Zhao, 2016; Ma & Xu, 2017; Xu, 2019, 2020a, 2021). The age of this biota (Pelsonian, Anisian, ~244.2 Ma) is well constrained by conodont biozonation and zircon dating (Zhang et al., 2009, 2015), and therefore this taxon documents the earliest known member of *Peltoperleidus*, predating the previously oldest record near the Anisian/Ladinian boundary (~242 Ma) from Europe by approximately two Ma. The fossil beds are composed of thinly laminated micritic limestones alternating with silty limestones, indicating a semi-enclosed intraplatform depositional environment in in the Triassic Yangtze Sea, a part of the eastern Palaeotethys Ocean (Hu et al., 2011; Metcalfe, 2011; Benton et al., 2013).

## Materials and Methods

The specimens are curated at the fossil collections of the Institute of Vertebrate Paleontology and Paleoanthropology (IVPP), Chinese Academy of Sciences in Beijing, China. They were prepared with sharp steel needles. For better contrast, some specimens were dusted with ammonium chloride (NH_4_Cl) before being photographed. The relative position of fins and scale counts were expressed following Westoll (1944). The traditional actinopterygian nomenclatures (Gardiner & Schaeffer, 1989; Bürgin, 1992; Grande & Bemis, 1998) are generally followed, for ease of comparison with most existing literature. The segmented and unbranched rays anterior to the principal fin rays are termed as procurrent rays, following Arratia (2008, 2009).

The new taxon was incorporated in a phylogenetic analysis based on the data matrix of Ma, Xu & Geng (2021). The current data matrix was generated by WinClada 1.00.08 (Nixon, 2002), including 134 morphological characters and 59 actinopterygian taxa (see Supplemental Material). The sampled taxa included almost all species of *Peltoperleidus* from Europe with an exception of *P. triseries* that is represented by a single incomplete specimen lacking phylogenetically important information on the skull. All characters were unordered and equally weighted. The basal actinopterygian *Moythomasia durgaringa* was selected for out-group comparison. Tree searches were accomplished with the heuristic search algorithm (gaps treated as missing data; 500 random addition sequence replicates; tree bisection-reconnection (TBR) branch-swapping, with five trees held at each step and multiple trees saved) in PAUP * 4.0b10 (Swofford, 2003).

The electronic version of this article in Portable Document Format (PDF) will represent a published work according to the International Commission on Zoological Nomenclature (ICZN), and hence the new names contained in the electronic version are effectively published under that Code from the electronic edition alone. This published work and the nomenclatural acts it contains have been registered in ZooBank, the online registration system for the ICZN. The ZooBank LSIDs (Life Science Identifiers) can be resolved and the associated information viewed through any standard web browser by appending the LSID to the prefix http://zoobank.org/. The LSID for this publication is: urn:lsid:zoobank.org:pub: F1B1BD3A-DD3F-4876-A7EE-649C8B224E31. The online version of this work is archived and available from the following digital repositories: PeerJ, PubMed Central and CLOCKSS.

## Results

**Systematic paleontology**

Actinopterygii Cope, 1887

Neopterygii Regan, 1923

Louwoichthyiformes Xu, 2021

**Emended diagnosis.** Stem group of neopterygians distinguished from other members of this clade by the following combination of features (autapomorphies identified with an asterisk): no more than two suborbitals confined between dermosphenotic and preopercle; maxilla relatively short and deep, ending nearly below posterior orbital margin; presence of teeth only on anterior half portions of both jaws; ventral portion of preopercle anterior extended, contacting maxilla anteriorly (*); posttemporal relatively narrow, not reaching median line; branchiostegal rays relatively few, two or three pairs in number; subopercle slightly larger than opercle, with prominent anteroventral extension (*).

Peltoperleididae fam. nov.

LSID urn:lsid:zoobank.org:act: 80D7CFFE-379A-4012-BC46-73D855D42BCA

**Type genus.***Peltoperleidus*Bürgin et al., 1991.

**Diagnosis.** As for the monotype genus.

**Geographical distribution and age.** As for the monotype genus.

*Peltoperleidus* Bürgin et al., 1991

**Emended Diagnosis.** Small-sized louwoichthyiforms distinguished from louwoichthyids and pseudobeaconiids by the following features (autapomorphies, those unique among louwoichthyiforms, identified with an asterisk): two or three horizontal rows of deepened scales (lateral line scales and one or two horizontal rows just ventral to them) in anterior flank region; fusion of paired frontals into single element; fusion of paired parietals into single element; presence of molariform teeth on prearticular and palatine bones; absence of epaxial procurrent rays in caudal fin (*); and presence of six or more epiaxial basal fulcra associated with caudal fin (*).

**Content.***Peltoperleidus ducanensis*Bürgin et al., 1991; *P. obristi*Herzog, 2001; *P. macrodontus* Bürgin, 2002; *P. triseries* Bürgin, 2002; *P. asiaticus* sp. nov.

**Type species.***Peltoperleidus ducanensis*Bürgin et al., 1991.

**Geographical distribution and age:** Besano, Italy; Monte San Giorgio and Ducantal, Valbellahorn, Switzerland; Luoping, Yunnan, China; Pelsonian (Anisian) to Ladinian, Middle Triassic.

*Peltoperleidus asiaticus* sp. nov.

LSID urn:lsid:zoobank.org:act: 97631528-FB7E-4B82-9FF8-33955D8E2DDE

(Figs. 1–9).

**Figure 2:**
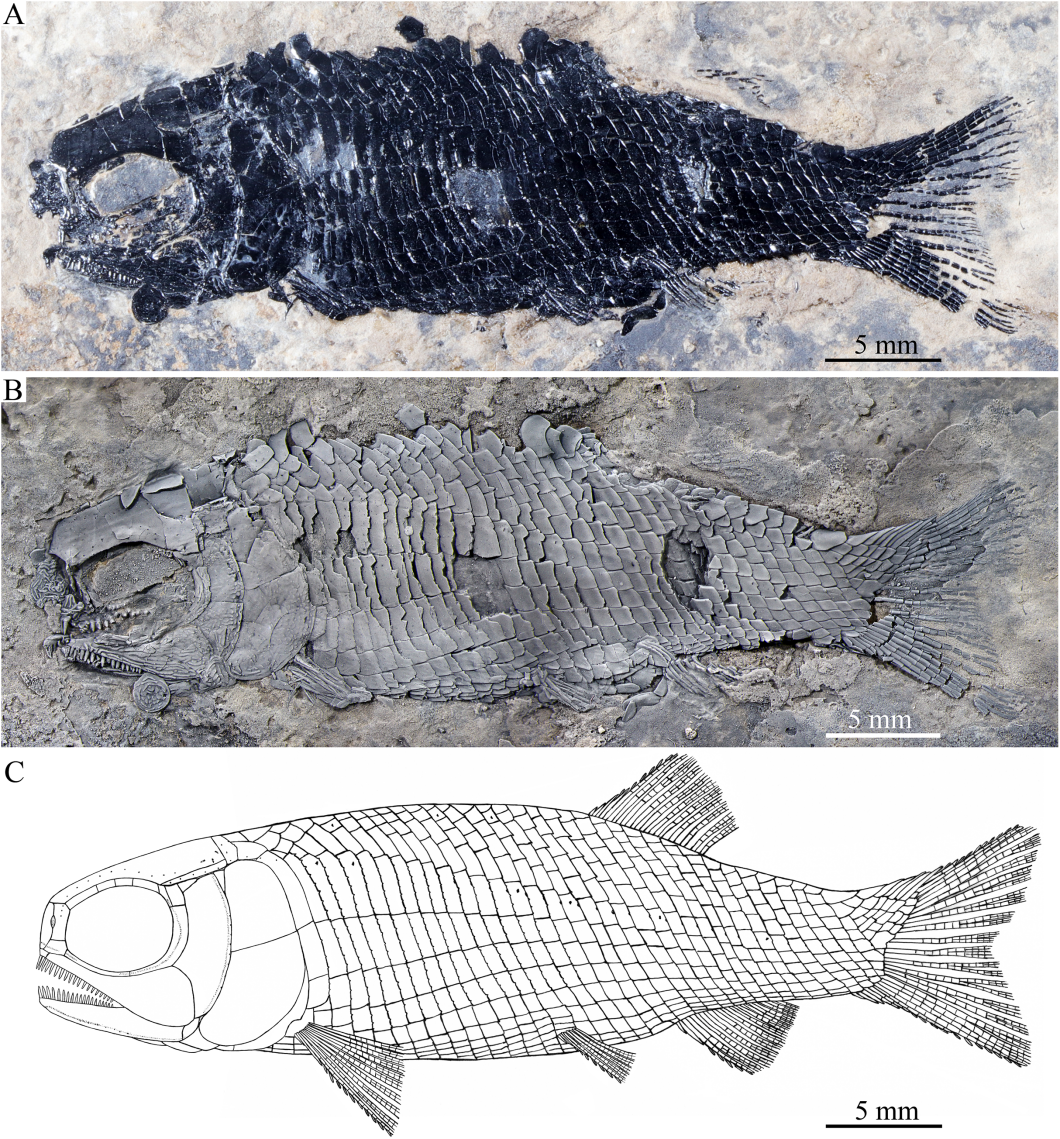
Holotype and reconstruction. Holotype and reconstruction of *Peltoperleidus asiaticus* sp. nov. (A) IVPP V22942, holotype. (B) Specimen dusted with ammonium chloride. (C) Reconstruction.

**Figure 3:**
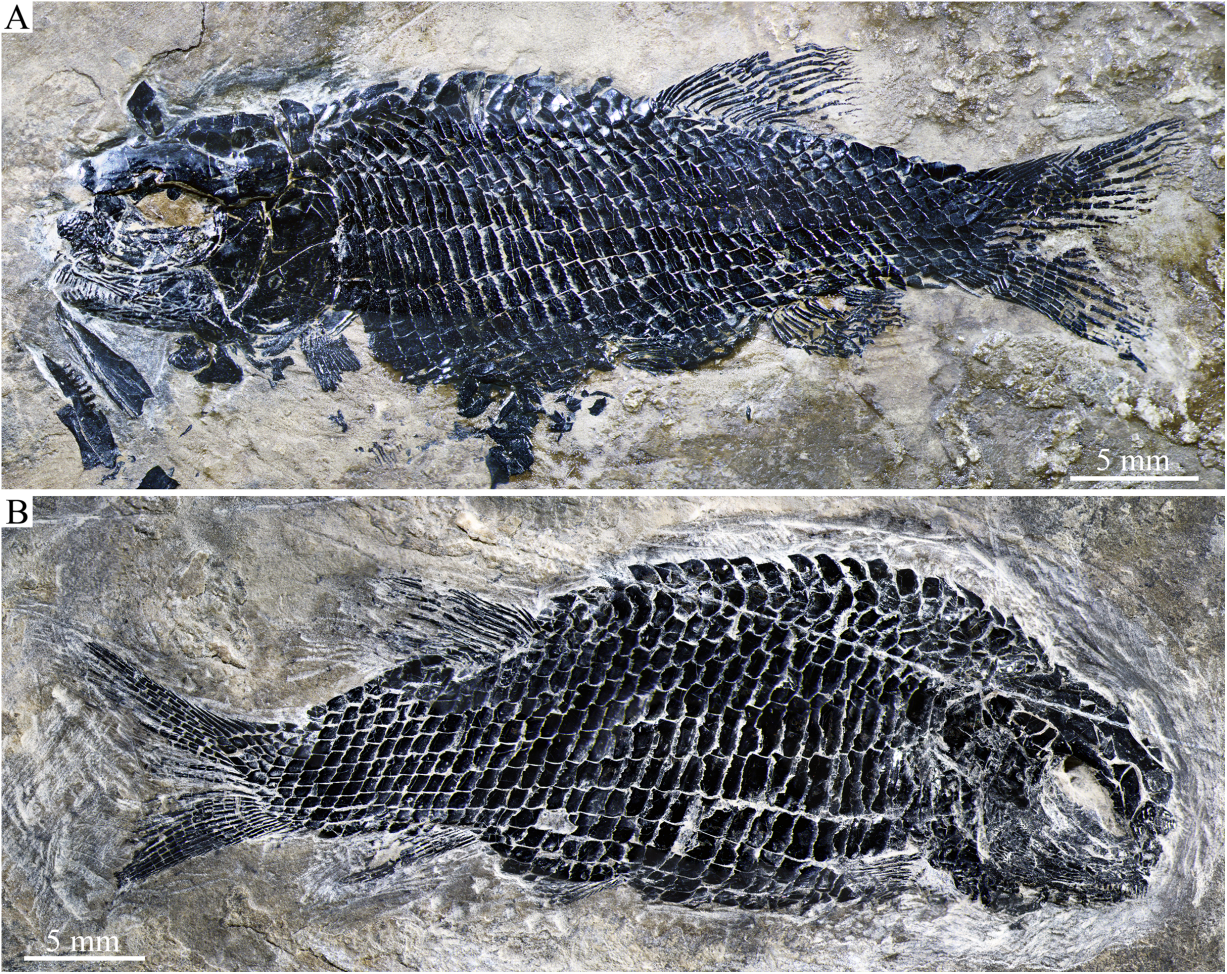
Two paratypes. Two paratypes of *Peltoperleidus asiaticus* sp. nov. (A) IVPP V25694. (B) IVPP V25695.

**Figure 4:**
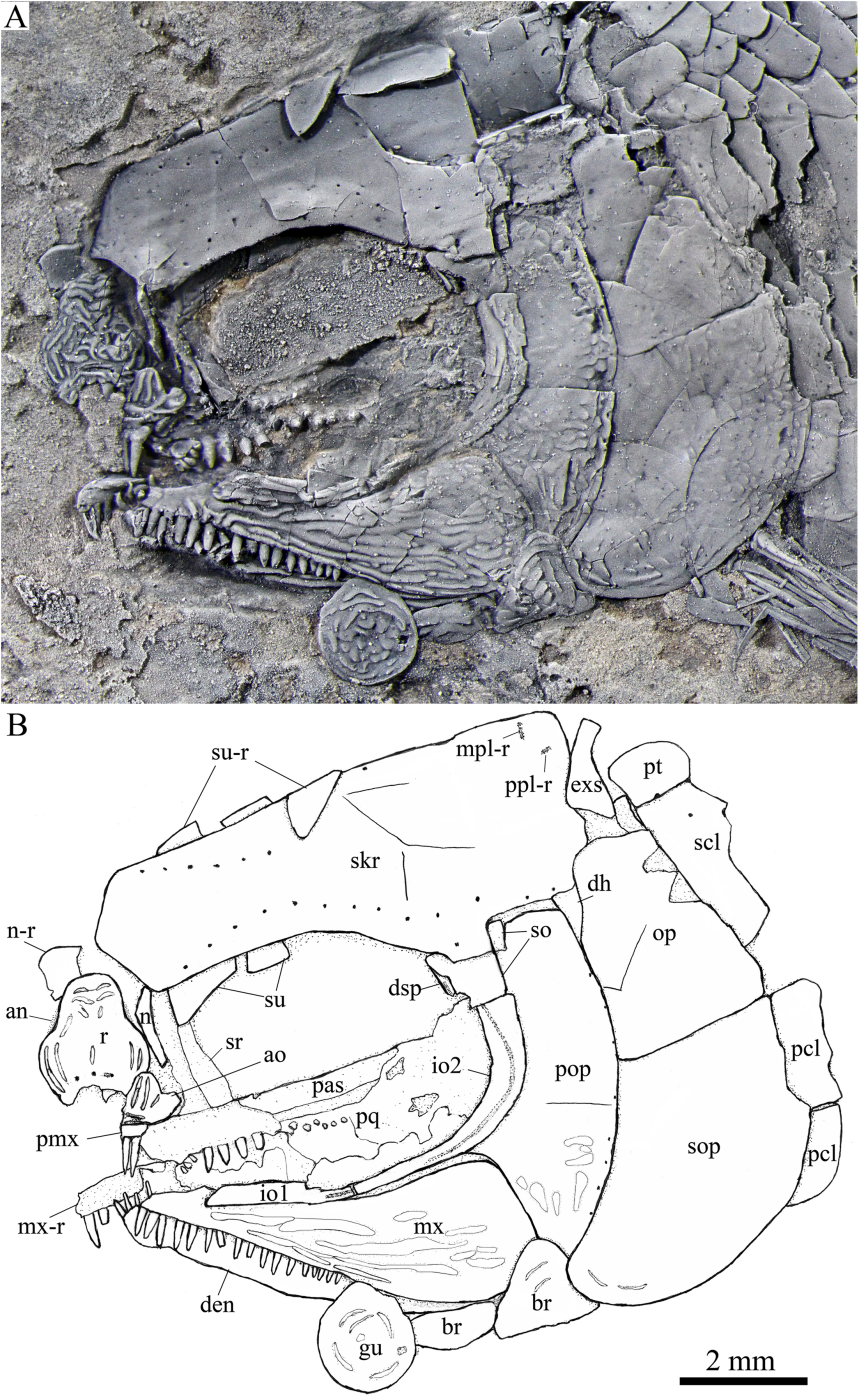
Skull and pectoral girdle in the holotype. Skull and pectoral girdle of *Peltoperleidus asiaticus* sp. nov., IVPP V22942 (holotype).

**Figure 5:**
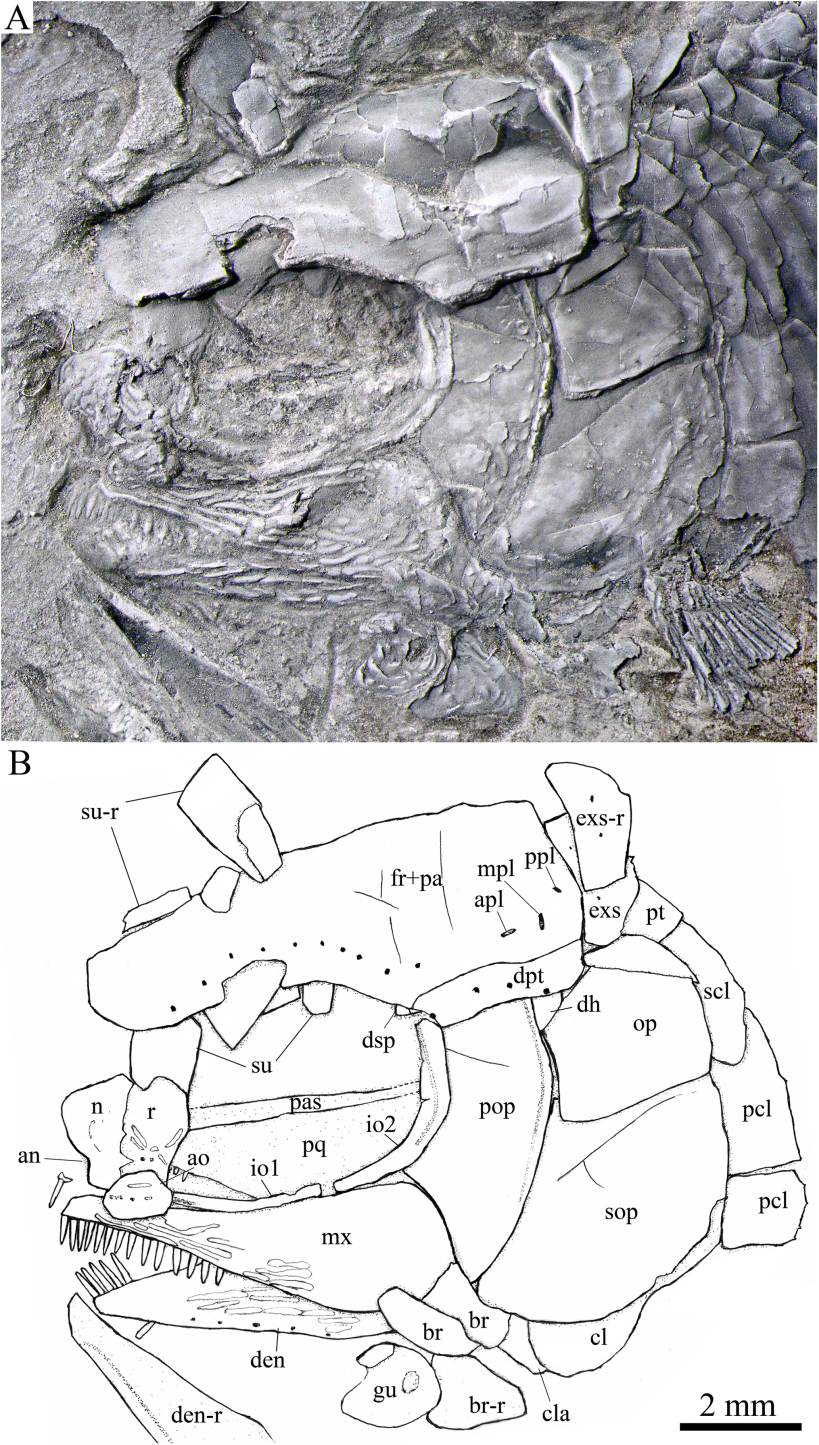
Skull and pectoral girdle in IVPP V25694. Skull and pectoral girdle of *Peltoperleidus asiaticus* sp. nov., IVPP V25694. (A) Photograph, dusted with ammonium chloride. (B) Line-drawing.

**Figure 6:**
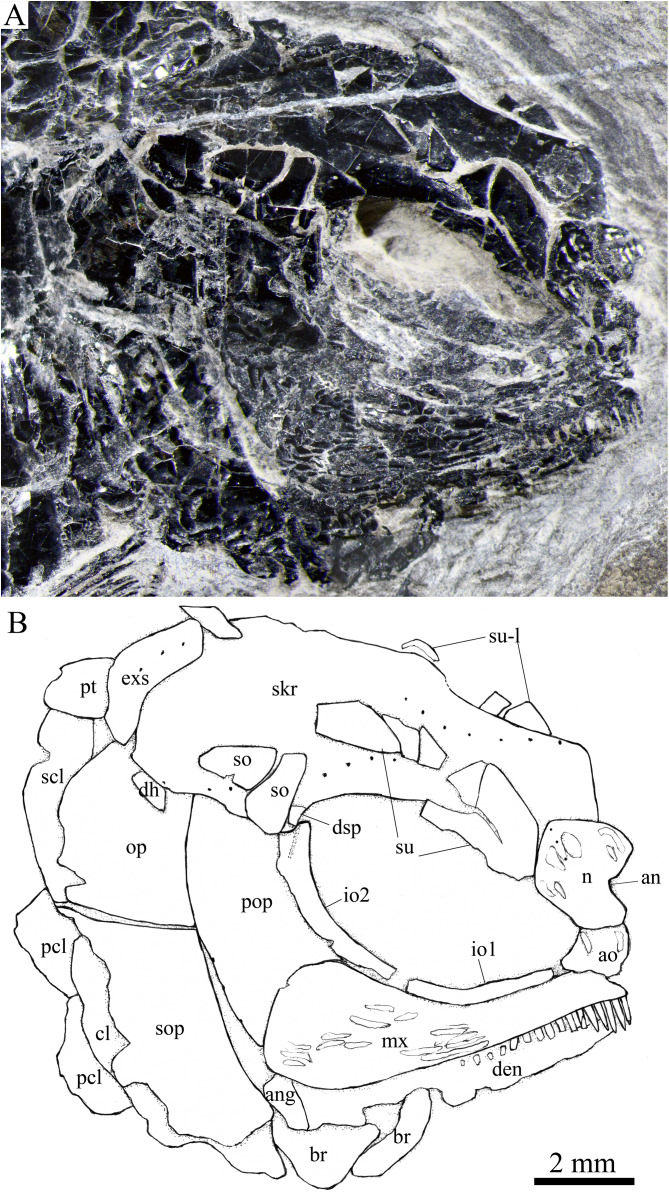
Skull and pectoral girdle in IVPP V25695. Skull and pectoral girdle of *Peltoperleidus asiaticus* sp. nov., IVPP V25695. (A) Photograph. (B) Line-drawing.

**Figure 7:**
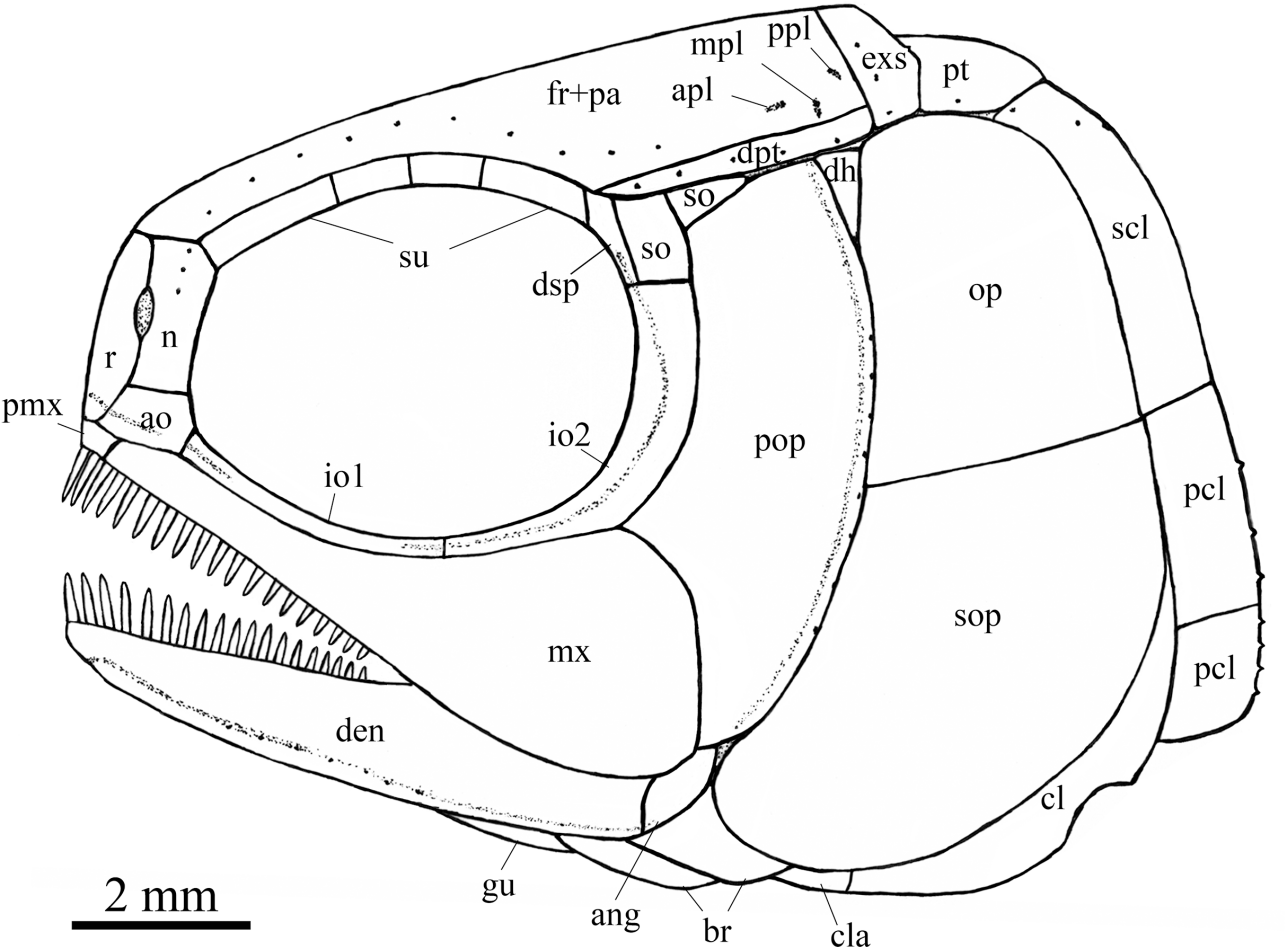
Reconstruction of skull and pectoral girdle. Reconstruction of skull and pectoral girdle of *Peltoperleidus asiaticus* sp. nov.

**Figure 8:**
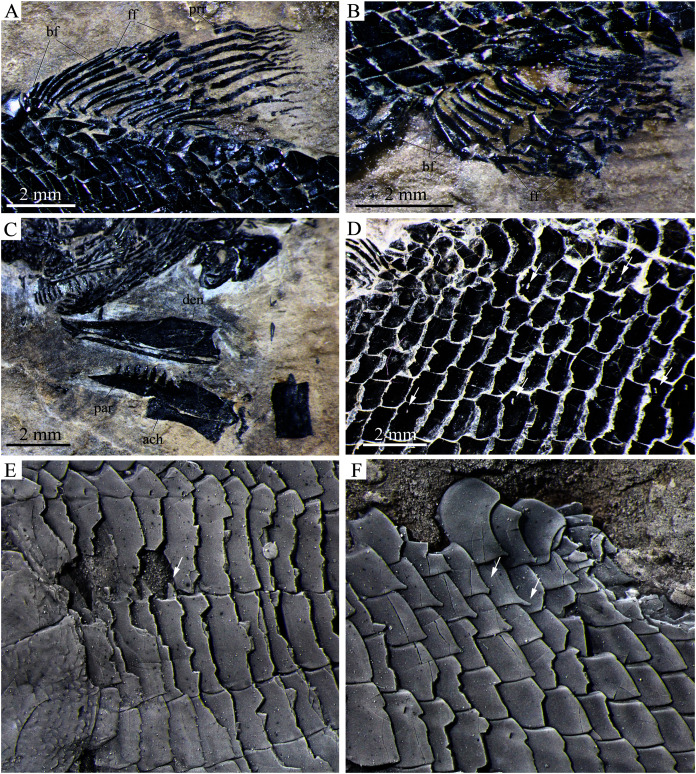
Detached cranial bones, fins and scales. Detached cranial bones, fins and scales of *Peltoperleidus asiaticus* sp. nov. (A–C) IVPP V25694; (A) Dorsal fin, (B) Anal fin, and (C) Dentary, prearticular and anterior ceratohyal. (D) IVPP V25695, flank scales, with arrows indicating pores in additional (upper) and main lateral line scales (below). (E, F) IVPP V22942, dusted with ammonium chloride; (E) Deepened anterior flank scales, with arrow indicating the dorsal peg of the scale, and (F) Scales with a prominent posteroventral spine in the predorsal region.

**Figure 9:**
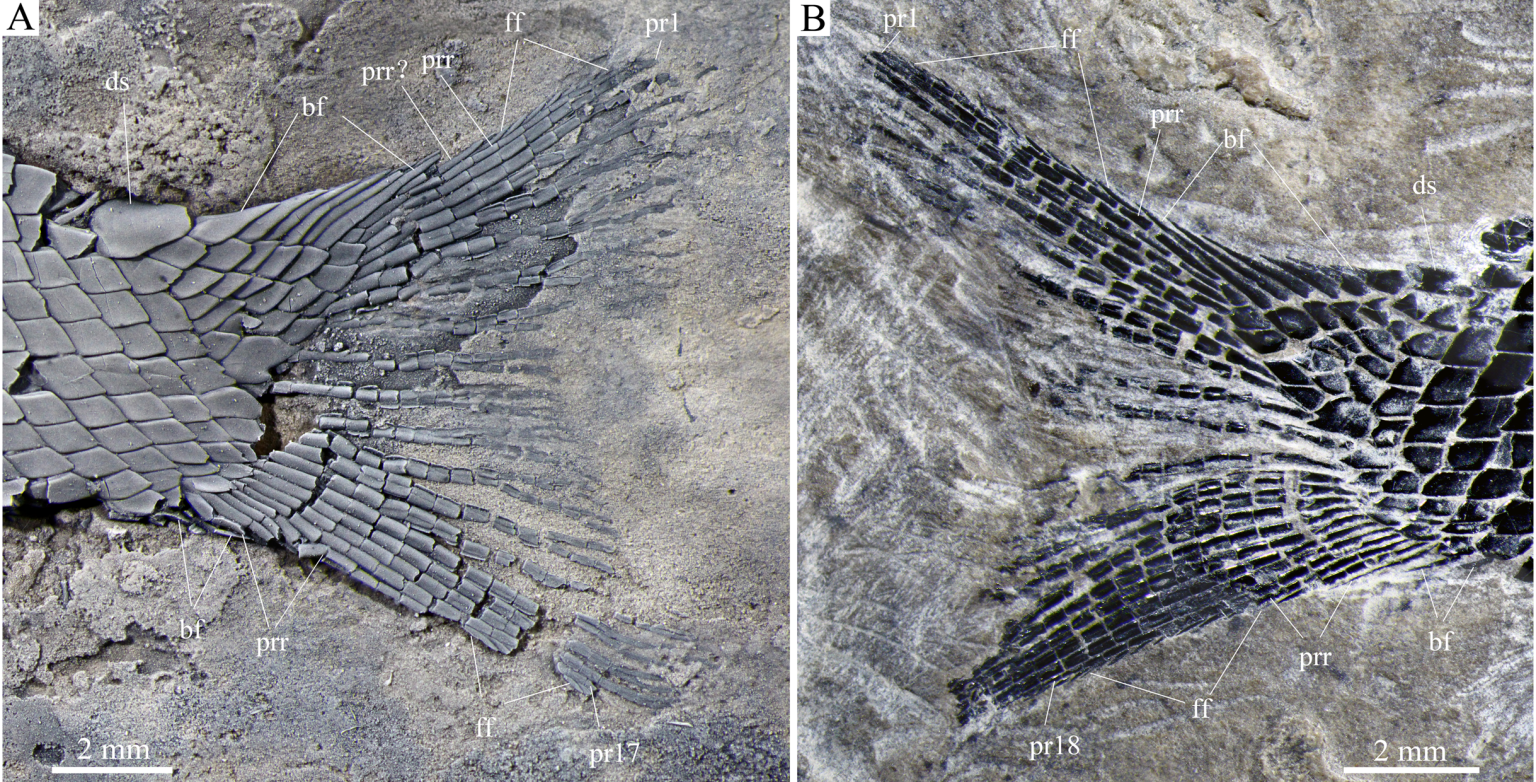
Caudal fin. Caudal fin of *Peltoperleidus asiaticus* sp. nov. (A) IVPP V22942 (holotype), dusted with ammonium chloride; (B) IVPP V25695.

**Etymology.** The specific epithet is derived from the Latin *asiaticus*, means Asia.

**Holotype.** IVPP V22942, a nearly complete specimen with part of left pectoral fin missing.

**Paratype.** IVPP V25694 and 25695.

**Locality and horizon:** Luoping, Yunnan, China; Second (Upper) Member of Guanling Formation, Pelsonian (~244.2 Ma), Anisian, Middle Triassic (Zhang et al., 2009).

**Diagnosis:** A new species of *Peltoperleidus* distinguished from other species of this genus by the following features (autapomorphies identified with an asterisk): rostral more expanded ventrally than dorsally, with width/length ratio of 0.8; frontals constricted above orbit, with narrowest width 26% of length of fused frontals and parietals (*); presence of four supraorbitals; presence of two suborbitals (*); premaxilla small, with two peg-like teeth; maxilla and dentary strongly ornamented with ganoid ridges; ten principal dorsal fin rays; 14 anal fin rays; 17 or 18 principal caudal fin rays; three horizontal rows of deepened scales in anterior flank region; anterior flank scales with serrated posterior margin; scales in predorsal region with prominent posteroventral spine (*), and pterygial formula of D22–23/P12–13, A19–21, C31–32/T34–35 (*).

**Description**

**General morphology and size.** Similar to other species of the genus, *Peltoperleidus asiaticus* sp. nov. is a small-sized stem-neopterygian fish with a blunt snout, a fusiform body and an abbreviated heterocercal caudal fin. The dorsal fin inserts slightly posterior to the origins of pelvic fins. The holotype (Figs. 2A, 2B) has a head length of 12 mm, a standard length (the length from the tip of the snout to the posterior extremity of the caudal peduncle) of 38 mm, and a total length of about 46 mm. The greatest body depth lies midway between the posterior margin of the opercle and the origin of the dorsal fin, being 13 mm in the holotype. The two paratypes have a standard length of 36 mm (Fig. 3A) and 38 mm (Fig. 3B), respectively. The body is fully covered with rhomboid scales, and the vertebral column and pterygiophores are not exposed.

**Snout.** The snout region is composed of a median rostral and a pair of nasals and antorbitals (Figs. 4–7). The rostral is broad and shield-like, more expanded ventrally than dorsally. The greatest width is about 80% of the depth of this bone. The ventral margin of the rostral is concave and the dorsal margin rounded. A distinct notch for the anterior nostril is present at the lateral margin. The anterior commissure of the lateral line system is enclosed in the rostral, indicated by a generally curved line of three small pores at the anteroventral portion of this bone. The outer surface of the rostral is ornamented with curved ridges and some tubercles.

The nasal is relatively large and irregular, bearing a large notch for the anterior nostril at its medial margin (Figs. 5, 6). It contacts the rostral medially, the frontal and first supraorbital dorsally, and the antorbital ventrally. The lateral margin of the nasal is slightly concave, forming a part of the anterior margin of the orbit. There is no distinct notch for the posterior nostril at the lateral margin of the nasal. The ornamentation of the nasal includes some ridges and tubercles. The supraorbital sensory canal extends into the nasal from the frontal, with a longitudinal line of three small pores discernable at the dorsal portion of the nasal (Fig. 5).

The antorbital is small and nearly trapezoidal, contacting the rostral anteromedially, the nasal dorsally, the premaxilla and maxilla ventrally, and the first infraorbital posteriorly (Figs. 4, 5). The posterolateral edge of the antorbital forms part of the anteroventral margin of the orbit. The ethmoid commissure between the rostral and antorbital bone is continuous. The outer surface of the antorbital is ornamented with ganoid ridges and tubercles.

**Skull roof.** The paired frontals, parietals and dermopterotics are fused into a broad skull roof plate in the holotype (Fig. 4) and IVPP V25695 (Fig. 6). In IVPP V25694 (Fig. 5), however, a suture is discernable between the left dermopterotic and the fused frontals and parietals. The skull roof plate tapers anteriorly to form a short, pointed process at the medial line of the skull. The frontal portion is constricted above the orbit, where the narrowest width is 26% of the length of the skull roof plate. The trajectory of the supraorbital canal can be traced by the openings of small pores into the smooth surface of the skull roof plate. Three short pit-lines are present in the parietal portion (Figs. 4, 5), including an anterior pit-line, a laterally extended middle one and a posterolaterlly extended posterior one. Indicated by a line of small pores, the temporal sensory canal runs longitudinally through the dermopterotic bone or the dermopterotic portion of the skull roof plate (Figs. 4–6).

The extrascapular is largely trapezoidal, having a concave anterior margin and a convex posterior margin. A small spine is discernable at the middle portion of the posterior margin of the left extrascapular in IVPP V25694 (Fig. 5). Each extrascapular tapers medially and contacts its counterpart at the mid-line of the skull. The supratemporal commissure transverses through the middle portion of the extrascapular (Figs. 5, 6).

**Circumorbital bones.** There are four rectangular or trapezoidal supraorbitals, and their sizes slightly vary in different specimens (Figs. 4, 5). Among them, the first (anterior-most) supraorbital is consistently the largest, the middle two are smallest, and the last is slighter shorter than the first.

Two infraorbitals are present (Figs. 4–6). The first is elongate and tube-like, with a concave dorsal margin and a convex ventral margin. The second is slightly expanded and curved, contacting the first anteriorly, the maxilla ventrally, the preopercle posteriorly, and the dermosphenotic and anterior suborbital dorsally. The dermosphenotic is small and narrow, tapering ventrally; its depth is nearly equal to the length of the last supraorbital. Posteriorly, two suborbitals are present (Figs. 4, 6). The anterior is relatively large and trapezoidal, 1.8 times deeper than long, and the posterior is smaller and nearly triangular, half of the depth of the anterior.

The infraorbital sensory canal passes longitudinally through the first infraorbital, extends upwards through the second infraorbital and enters the dermosphenotic. Part of narrow and slightly curved sclerotic bone is discernable near the anterior orbital margin in the holotype (Fig. 4), but its complete shape is still unknown because of incomplete preservation.

**Upper jaw.** The upper jaw consists of premaxilla and maxilla. The premaxilla is quite small, bearing two long, peg-like teeth on its oral margin (Fig. 4). The maxilla has a slender anterior ramus and a posterodorsally expanded cheek plate; the ratio of the length to the maximum depth of the maxilla is 2.8 in the holotype. The posterior margin of the maxilla is nearly rounded, ending at the level of the posterior margin of the second infraorbital. The outer surface of the maxilla is strongly ornamented with ganoid ridges. The oral margin of the maxilla is slightly convex, bearing 17 peg-like teeth on its anterior portion. The anterior teeth are longest and slightly recurved distally with an acuminate acrodine apex; the teeth gradually reduce in length posteriorly.

The lower jaw is wedge-shaped, ornamented with longitudinal ridges. Two elements, dentary and angular, are discernable in lateral view (Figs. 6, 7). Additionally, a prearticular is well exposed in medial view (Fig. 8C). The dentary is largest, accounting for 92% of the length of the lower jaw. The angular is small and narrow, contacting the dentary posteriorly. The prearticular is slightly shorter than the dentary, having a pointed anterior tip, a curved posterior margin, a convex dorsal margin and a nearly straight ventral margin. The mandibular canal runs longitudinally through the dentary parallel to the slightly convex ventral margin of this bone and enters the angular posteriorly. The posterodorsal portion of the lower jaw is laterally covered by the maxilla; the supra-angular, commonly present in other stem-neopterygians, is not exposed. The teeth in the dentary are peg-like, similar to those of the maxilla in size. The anterior teeth are longest and slightly inclined anteriorly, and others gradually reduce in length posteriorly. A row of seven teeth is present in the oral margin of the prearticular (Fig. 8C). The teeth are molariform, stronger and blunter than those in the dentary; the fifth teeth are longest, and the last shortest with a rounded tip.

**Parasphenoid and palatoquadrate.** The elongate parasphenoid and plate-like palatoquadrate are partly discernable through the orbit, but no distinct palatine bones can be identified (Figs. 4, 5). A series of teeth are discernable in the medial margin of the palatine. The teeth are large and molariform, shorter but stronger than those in the maxilla.

**Opercular series and dermohyal.** The preopercle is sickle-shaped, tapering ventrally. Anteriorly, it has a short triangular process inserting between the second infraorbital and the maxilla (Figs. 4–6). The ornamentation of the preopercle includes some short ridges on its posterior and ventral parts (Fig. 4). The preopercular sensory canal is indicated by a vertical line of small pores close to the posterior margin of this bone.

Both the opercle and subopercle are trapezoidal. The opercle is relatively small, with a depth/length ratio of 1.3. The subopercle is larger, 1.3 times as deep as the opercle, having a prominent anteroventral extension that reaches the posterior end of the lower jaw (Fig. 6). In addition, a small triangular dermohyal is wedged between the preopercle and opercle. The opercle and dermohyal are nearly smooth on their surfaces, and the subopercle is ornamented with some short ridges. An interopercle is primitively absent, as in other stem neopterygians.

**Gular, branchiostegal rays and ceratohyal.** The median gular is nearly drop-shaped, with a slightly pointed anterior tip and a rounded posterior margin. It has a length about 27.5% of that of the maxilla. Two pairs of branchiostegal rays are present (Figs. 4–6); the anterior are small and elongate, and the posterior larger and triangular. The outer surfaces of the gular and branchiostegal rays are ornamented with ganoid ridges and tubercles.

A detached anterior ceratohyal is preserved near the prearticular (Fig. 8C). It is hourglass-shaped, half of the length of the dentary. The posterior ceratohyal and other elements of the hyoid series are not exposed.

**Paired girdles and fins.** A posttemporal, a supracleithrum, a cleithrum, a clavicle and two postcleithra are present on each side of the pectoral girdle (Figs. 4–7). The posttemporal is triangular, half as wide as the extrascapular. The supracleithrum is rhomboidal, nearly as deep as the opercle. The lateral line pierces the lateral portion of the posttemporal and runs posteriorly through the dorsal portion of the supracleithrum (Fig. 4).

Most of the curved cleithrum is laterally covered by the subopercle and its complete shape remains unknown. A triangular clavicle contacts the anteroventral arm of the cleithrum. Additionally, there are two rhomboidal postcleithra associated with the cleithrum; the dorsal is twice as deep as the ventral. Several small spines are discernable at the posterior margins of both postcleithra (Fig. 5).

The pectoral fins insert low on the body, and each is composed of about ten distally segmented rays, preceded by two basal fulcra and a series of small leaf-like fringing fulcra (Figs. 3, 5A). The pelvic girdles are not exposed. The pelvic fins are small, located at the 12^th^ or 13^th^ vertical scale row. Each has five distally segmented rays, preceded by three basal fulcra and a series of fringing fulcra.

**Median fins.** The dorsal fin originates above the 22^nd^ or 23^rd^ vertical scale row, including a procurrent ray and ten principal rays (Fig. 8A). All rays are distally segmented. The procurrent ray is unbranched, preceded by four basal fulcra and a series of fringing fulcra. The first principal ray is unbranched and longest, and others are branched distally and gradually reduce in length posteriorly.

The anal fin originates below the 19^th^ to 21^st^ vertical scale row, and is composed of 14 distally segmented principal rays (Fig. 8B). The first ray is unbranched, preceded by two basal fulcra and a series of fringing fulcra; the remaining rays are branched distally.

The caudal fin is abbreviated heterocercal with a forked posterior profile. It is composed of 17 or 18 principal rays, eight in the dorsal lobe (Fig. 9). In addition, one or two procurrent rays are present at the dorsal lobe, and three or four procurrent rays at the ventral lobe. All rays are smooth on their surfaces. Both marginal principal rays are unbranched, and the rest branched distally. Small leaf-like fringing fulcra are associated with the leading margin of the procurrent ray in the dorsal lobe, and with leading margins of the 17^th^ or 18^th^ principal ray and last procurrent ray in the ventral lobe (Fig. 9B). There are six epaxial basal fulcra and two hypaxial basal fulcra; they are elongate, leaf-like elements. An anteriorly expanded dorsal scute precedes the anterior epaxial basal fulcrum, and a ventral scute is absent.

**Scales.** The body is fully covered with rhomboidal scales. The scales are smooth, arranged in 34 or 35 vertical rows between the pectoral girdle and the caudal inversion. On each side of the body, there are 14 or 15 scales in the 22^nd^ vertical row, six or seven above the lateral line. The lateral line scales and two horizontal rows of scales immediately ventral to them are notably deepened in the anterior flank region. Each lateral line scale has a small notch at its posterior margin (Fig. 8F). The anterior lateral line scales are deepest, nearly three times as deep as long, and the depths of the lateral line scales gradually become shorter posteriorly. A small pore is present at the dorsal portion of some lateral line scales. Moreover, several small pores on the scales in the pre-dorsal region indicate the presence of an additional lateral line (Fig. 8D). The scales in anterior flank region have a serrated posterior margin, whereas those in other regions are nearly straight in their posterior margins. Peg-socket articulations are discernable between scales in the anterior flank region (Fig. 8E). Additionally, some scales in the pre-dorsal region bear a prominent posteroventral pine (Fig. 8F).

## Discussion

### Phylogenetic relationships

**Figure 10:**
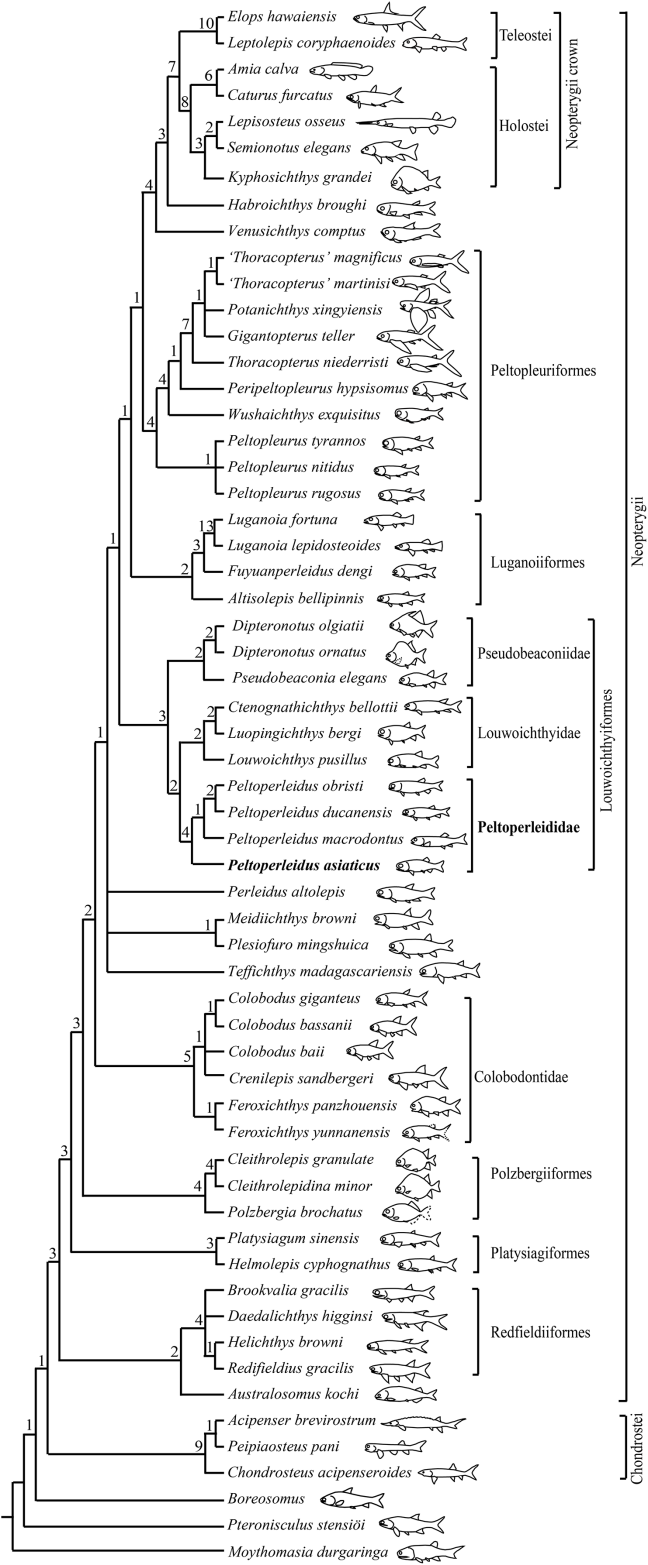
Strict consensus of 120 most parsimonious trees. Strict consensus of 120 most parsimonious trees (tree length = 384 steps, consistency index = 0.4505, retention index = 0.7719), illustrating the phylogenetic position of *Peltoperleidus asiaticus* sp. nov. within the Neopterygii. Digits above nodes indicate Bremer decay indices. For character descriptions and data matrix, see the online Supplemental Material.

My analysis resulted in 120 most parsimonious trees (tree length = 384 steps, consistency index = 0.4505, retention index = 0.7719). The strict consensus tree (Fig. 10) shows that the traditional, broadly inclusive ‘Perleidiformes’ or even ‘Perleididae’ are paraphyletic groups composed of a series of independent stem-neopterygian lineages, in accordance with other recent analyses (Xu, Gao & Coates, 2015; Xu, Zhao & Shen, 2015, Xu, Ma & Zhao, 2018; Wen et al., 2019; Xu, 2020a, 2021; Ma, Xu & Geng, 2021). In this cladogram, four species of *Peltoperleidus* form a well-supported monophyletic group within the Louwoichthyiformes, and *P. asiaticus* sp. nov. is recovered at the base of *Peltoperleidus*.

*Peltoperleidus* is referred to the Louwoichthyiformes because it shares four synapomorphies of this order: the ventral portion of the preopercle anterior extended, contacting maxilla anteriorly (rather than ventrally); maxilla relatively short, ending nearly below the posterior margin of the orbit; presence of a prominent anteroventral extension of the subopercle; and presence of two or three pairs of branchiostegal rays. Within the Louwoichthyiformes, *Peltoperleidus* is more closely related to louwoichthyids than to pseudobeaconiids, sharing three derived features with louwoichthyids: presence of a small premaxilla with only one or two marginal teeth (reverse in *P. obristi* and *P. ducanensis*); presence of only two pairs of branchiostegal rays; and presence of a triangular posterior-most branchiostegal ray.

Four species of *Peltoperleidus* form a monophyletic group, supported by four synapomorphies: fusion of paired frontals into a median bone, fusion of paired parietals into a median bone, absence of epaxial procurrent rays in the caudal fin, and presence of six or more epiaxial basal fulcra associated with the caudal fin. *P. asiaticus* sp. nov. is recovered at the base of *Peltoperleidus* because it possesses the synapomorphies of this genus listed above but lacks the derived feature of *P. macrodontus* shared with *P. ducanensis* and *P. obristi*, presence of two horizontal rows of notably deepened scales (lateral line scales and the scales just ventral to them) in anterior flank region (independently evolved in *Altisolepis*). Above *P. macrodontus*, the sister taxon relationships between *P. ducanensis* and *P. obristi* are supported by presence of the supraorbital sensory canal ending in the frontal, and presence of a relatively long premaxilla with three or more teeth (independently evolved in *Pseudobeaconia*).

### Peltoperleididae, a new family of Louwoichthyiformes

I propose a new family (Peltoperleididae) for *Peltoperleidus* within Louwoichthyiformes because the family possesses diagnostic features of this order but shows distinct morphological gaps with other louwoichthyiforms (Xu, 2021). Peltoperleidids lack two derived features of pseudobeaconiids, including presence of only one or two supraorbital bones and presence of a posteriorly directed spine on ridge scales anterior to the dorsal fin. Additionally, peltoperleidids lack two derived features of louwoichthyids, presence of extraordinarily long, fang-like marginal teeth on jaws and the absence of molariform teeth on coronoids, prearticular and palatine. These dentation differences could implicate disparity of feeding between peltoperleidids and louwoichthyids. The feeding apparatus of louwoichthyids are adapted for biting; they could use their long and sharp teeth to grasp and tear the prey from the substrate or to bite a piece from a larger prey item (Bürgin, 1996; Xu, 2021). However, peltoperleidids likely evolved a durophagous diet with dentition combining grasping and crushing morphologies (as described above), resembling colobodontids and some perleidids.

Notably, peltoperleidids are easily distinguished from other louwoichthyiforms in the skull roof. The frontals and parietals are consistently fused into median bones in all species of *Peltoperleidus*, contrasting the paired conditions in other louwoichthyiforms. A rudimental, transverse suture is present between the median frontal and parietal in three species of *Peltoperleidus* from Europe, but it is absent in *P. asiaticus*, in which the median frontal is further fused with the median parietal (with only a longitudinal suture between parietal and dermopterotic in the skull roof). Among other stem-neoptergyians, a median frontal is independently evolved in luganoiids and some peltopleuriforms (Bürgin, 1992; Xu, 2020a, 2020b).

Additionally, peltoperleidids are unique among louwoichthyiforms in lacking any epaxial procurrent rays in the caudal fin. By contrast, pseudobeaconiids and louwoichthyids, resembling many other stem-neopterygian clades, have three or more epaxial procurrent rays in the caudal fin. Outside louwoichthyiforms, the epaxial procurrent rays were independently lost in the *Plesiofuro-Meidiichthys* clade and Platysiagiformes (Wen et al., 2019; Xu, 2020a). In line with the absence of epaxial procurrent rays, peltoperleidids have at least six epiaxial basal fulcra associated with the caudal fin, contrasting other louwoichthyiforms that generally have three or four epiaxial basal fulcra in the caudal fin.

Moreover, peltoperleidids have two or three horizontal rows of notably deepened flank scales. *Peltoperleidus asiaticus* sp. nov. and *P. triseries* primitively have three rows of deepened flank scales, and derived peltoperleidids generally have two rows of even notably deepened flank scales, showing a condition similar to that of *Altisolepis*. The recovered basal position of *P. asiaticus* within *Peltoperleidus* in my analysis further supports that the presence of two horizontal rows of notably deepened scales was independently evolved in *Altisolepis* and peltoperleidids (Mutter & Herzog, 2004). By contrast, the lateral line scales are similar in depth to adjacent scales in other louwoichthyiforms.

### Validity and taxonomy of *Altisolepis*

My comparative study supports the validity of *Altisolepis* (Mutter & Herzog, 2004). *Altisolepis* differs from *Peltoperleidus* in the following features: a subopercle smaller than the opercle (larger than the opercle in *Peltoperleidus*); a long maxilla extending well posterior to the orbital margin (relatively short in *Peltoperleidus*); presence of teeth in almost full length of the oral margin of the maxilla (only anterior half in *Peltoperleidus*); presence of multiple epaxial procurrent rays in the caudal fin (absent in *Peltoperleidus*); presence of only two or three epaxial basal fulcra in the caudal fin (six or more in *Peltoperleidus*); presence of six pairs of branchiostegal rays (only two pairs in *Peltoperleidus*); and absence of fringing fulcra (present in *Peltoperleidus*). Based on the presence of deepened flank scales, Sun et al. (2015) placed *Altisolepis* in the Peltopleuridae (Peltopleuriformes). However, the greatly deepened scales persist to the caudal peduncle in *Altisolepis* but they are only present in the anterior flank region of peltopleuriforms. *Altisolepis* lacks the diagnostic features of Peltopleuriformes (senus Xu & Ma, 2016), *e.g*., supraorbital sensory canal ending in the frontal, absence of a preopercle/dermopterotic contact, presence of a postspiracle, and presence of enlarged lateral scutes associated with the anal fin. The absence of fringing fulcra in all fins support that *Altisolepis* is more closely related to the Fuyuanperleididae-Luganoiidae clade than to the Peltopleuriformes. Consequently, this genus is tentatively placed incertae sedis among the expanded Luganoiiformes (Fig. 10). A detailed taxonomic revision of *Altisolepis* will be undertaken in the future on the basis of comparative studies of *A. sinensis* (Sun et al., 2015) with its relatives from Europe (Bürgin, 1992; Mutter & Herzog, 2004).

### Biogeographic implications

The discovery of *Peltoperleidus asiaticus* sp. nov. in South China documents the first record of the genus outside Europe, and predates the previously oldest record near the Anisian/Ladinian boundary (~242 Ma) in Switzerland and Italy by approximately two Ma. During the Middle Triassic, South China was located at the eastern rim of the Palaeotethys Ocean, and Switzerland and Italy at the western rim of that ocean (Metcalfe, 2011). The older age and basal position of *P. asiaticus* within the genus indicates that *Peltoperleidus* probably originated in the early Middle Triassic of South China, and the Palaeotethys Ocean would have provided an east–west corridor for dispersal of the genus into Europe. The biological exchanges between the eastern and western rims of the Palaeotethys Ocean have previously been implicated by studies of other groups of ray-finned fishes and marine reptiles (Lombardo et al., 2011; Xu et al., 2012, Xu, Ma & Zhao, 2018; Benton et al., 2013; Sun et al., 2015; Wen et al., 2019; Xu, 2020a, Xu, 2021). The discovery of *P. asiaticus* provides an additional, new evidence supporting these exchanges in the Middle Triassic.

## Conclusion

The recovery and study of *Peltoperleidus asiaticus* sp. nov. from the early Middle Triassic (Anisian) of eastern Yunnan, South China adds our knowledge of the phylogeny and distribution of the genus and taxonomic diversity of early neopterygians in the Luoping Biota. Although small-sized, *P. asiaticus* is likely a durophagous predator with dentition combining grasping and crushing morphologies. The new species resembles *P. triseries* in primitively having three horizontal rows of deepened flank scales; the presence of two horizontal rows of deepened scales in other peltoperleidids are considered derived (independently evolved in *Altisolepis*). Phylogenetic studies support the validity of *Altisolepis* and its affinities with the Fuyuanperleididae-Luganoiidae clade, challenging the previous placement of the genus in the Peltopleuriformes. *Peltoperleidus* shares certain derived features with louwoichthyids, but its morphology is distinct enough from that of louwoichthyids, to warrant the erection of a new family (Peltoperleididae) within the Louwoichthyiformes. The older age and basal position of *P. asiaticus* implicate that the Peltoperleididae was probably originated in the early Middle Triassic of South China, and the Palaeotethys Ocean would have provided an east–west corridor for dispersal of this clade into Europe.

## Supplemental Information

10.7717/peerj.12225/supp-1 Supplemental Information 1 Supplementary figure, character list and data matrix.

10.7717/peerj.12225/supp-2 Supplemental Information 2 Data matrix.

## Abbreviations

ach: anterior ceratohyal
an: anterior nostril
ao: antorbital
apl: anterior pit line
bf: basal fulcrum
br: branchiostegal ray
cl: cleithrum
cla: clavicle
den: dentary
dh: dermohyal
dpt: dermopterotic
dsp: dermosphenotic
exs: extrascapular
ff: fringing fulcrum
fr: frontal
gu: gular
io: infraorbital
mpl: middle pit line
mx: maxilla
n: nasal
op: opercle
pa: parietal
par: prearticular
pas: parasphenoid
pcl: postcleithrum
ppl: posterior pit line
pr: principle fin ray
pmx: premaxilla
pop: preopercle
pt: posttemporal
pq: palatoquadrate
r: rostral
prr: procurrent ray
scl: supracleithrum
skr: skull roof plate
so: suborbital
sop: subopercle
sr: sclerotic bone
su: supraorbital

## Additional Information and Declarations

### Data Availability

The following information was supplied regarding data availability: The raw data are available in the Supplementary File. The specimens (IVPP V22942, 25694 and 25695) illustrated and described are stored at the fossil collections of the Institute of Vertebrate Paleontology and Paleoanthropology, Chinese Academy of Sciences in Beijing, China.

### New Species Registration

The following information was supplied regarding the registration of a newly described species: Publication LSID: urn:lsid:zoobank.org:pub:F1B1BD3A-DD3F-4876-A7EE-649C8B224E31 Family, Peltoperleididae Xu LSID: urn:lsid:zoobank.org:act:80D7CFFE-379A-4012-BC46-73D855D42BCA Species, Peltoperleididae Xu LSID: urn:lsid:zoobank.org:act:97631528-FB7E-4B82-9FF8-33955D8E2DDE.
